# Levels of Autonomous Radiology

**DOI:** 10.2196/38655

**Published:** 2022-12-07

**Authors:** Suraj Ghuwalewala, Viraj Kulkarni, Richa Pant, Amit Kharat

**Affiliations:** 1 DeepTek Medical Imaging Pvt Ltd Pune India; 2 Dr DY Patil Hospital DY Patil University Pune India

**Keywords:** artificial intelligence, automation, machine learning, radiology, explainability, model decay, generalizability, fairness and bias, distributed learning, autonomous radiology, AI assistance

## Abstract

Radiology, being one of the younger disciplines of medicine with a history of just over a century, has witnessed tremendous technological advancements and has revolutionized the way we practice medicine today. In the last few decades, medical imaging modalities have generated seismic amounts of medical data. The development and adoption of artificial intelligence applications using this data will lead to the next phase of evolution in radiology. It will include automating laborious manual tasks such as annotations, report generation, etc, along with the initial radiological assessment of patients and imaging features to aid radiologists in their diagnostic and treatment planning workflow. We propose a level-wise classification for the progression of automation in radiology, explaining artificial intelligence assistance at each level with the corresponding challenges and solutions. We hope that such discussions can help us address challenges in a structured way and take the necessary steps to ensure the smooth adoption of new technologies in radiology.

## Introduction

Advancements in artificial intelligence (AI) and machine learning have enabled the automation of time-consuming and manual tasks across different industries [1]. With substantial developments in the digital acquisition of data and improvements in machine learning and computing infrastructures, AI applications are also expanding into disciplines that were previously considered the exclusive province of human expertise [2]. From automobiles to the health care sector, the world is actively adopting AI to transform these respective industries.

The confluence of information and communication technologies with automotive technologies has resulted in vehicle autonomy. This growth is expected to continue in the future due to increasing consumer demand, reduction in the cost of vehicle components, and improved reliability [3]. The Society of Automotive Engineers has classified the progression of driving automation into 6 levels [4], ranging from *No Automation* (Level 0) to *Full Automation* (Level 5). The levels of driving automation are characterized by the specific roles played by each of the 3 principal players, that is, the human user (driver), the driving automation system, and other vehicle components. As vehicle autonomy increases with each level of automation, driver intervention is reduced [4].

Similar to the automobile industry, AI is progressively transforming the landscape of health care and biomedical research. A simulated deployment of natural language processing–based classification algorithm has been shown to enable automated assignment of computed topographic and magnetic resonance radiology protocols with minimal errors, resulting in a high-quality and efficient radiology workflow [5]. More recently, applications of diagnostic imaging systems have expanded the capabilities of AI in the previously unexplored and more complex health care sector [2]. In radiology, AI applications are being widely adopted for assisted image acquisition, postprocessing, automated diagnosis, and report generation. Automation in this field is still in its infancy, and several clinical and ethical challenges must be addressed before further progress can be made [6].

In this perspective, we attempt to categorize and map the advancements and challenges of automation in radiology into 6 levels, similar to driving automation, with radiologists, AI systems, and advanced technologies playing important roles at each level. The subsequent parts of the paper briefly discuss each level, its technical challenges, plausible solutions, and enabling factors required for transitioning into the next level.

## Levels of Automation in Radiology

**Figure 1:**
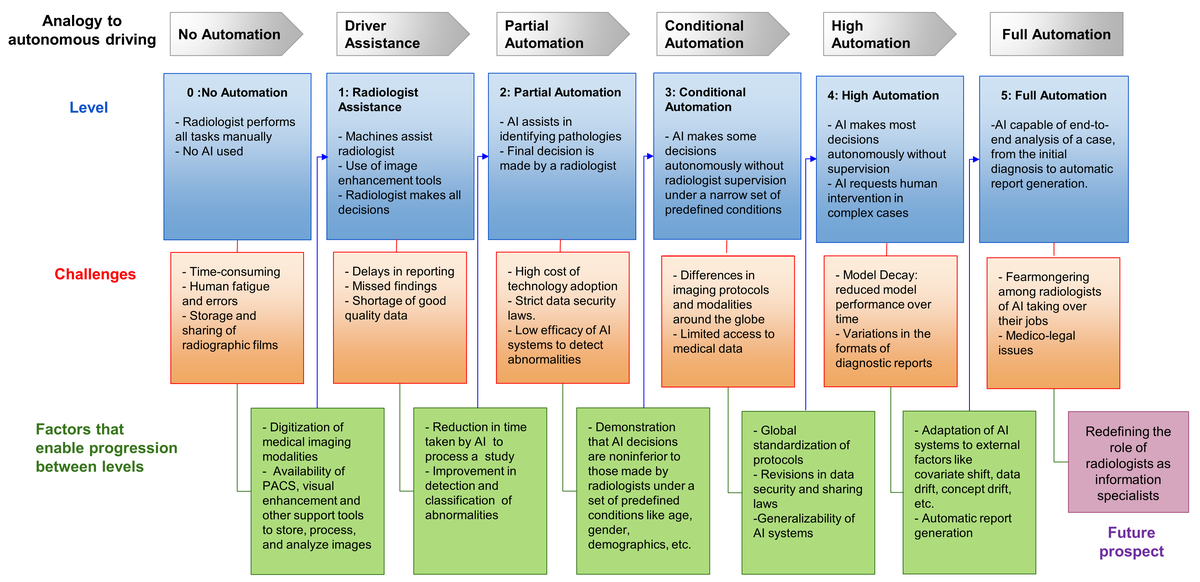
Flowchart depicting the various levels of automation in radiology practice. At each level, the role of the radiologist and artificial intelligence (AI) is outlined, along with the enabling factors required to mitigate the potential challenges for progression to the next level. PACS: picture archiving and communication systems.

The advancement of AI in the health sector has substantially bridged the gap between computation and radiology, paving the way for automation in radiology practice. We describe the 6 levels of automation in radiology using a taxonomy similar to that used in driving automation. We further attempt to provide a futuristic vision of the challenges that the radiology field may encounter as we progress toward the complete automation of this field. Figure 1 illustrates different levels of automation in radiology, including the challenges at each stage and the factors that enable the progression between levels.

## Level 0: No Automation

*Level 0,* also known as *No Automation*, is the stage where a radiologist manually performs every task from image acquisition and radiographic film processing to diagnostic analysis without the assistance of AI. We are well past this stage as the recent advances in medical imaging modalities have enabled digital storage and processing of the scans along with some automated assistance to aid in the imaging workflow.

## Level 1: Radiologist Assistance

At Level 1 automation, a radiologist performs most tasks manually with assistance from machines. Recent technological advancements have digitized medical scans, making it easier for radiologists to store, maintain, and distribute data. Furthermore, newer solutions include features such as contrast-brightness adjustment, assisted stitching of scans, assisted focus adjustment, etc, which simplify the imaging workflow and enable detailed radiological analysis. With everything digitized, these modalities generate enormous amounts of data, and the biggest challenge at this stage is the proper maintenance and storage of data [7]. This is where technologies such as picture archiving and communication systems have provided an economical solution to compress and store data for easy retrieval and sharing [8]. With the advancement in automation, the radiology field is currently experiencing a major paradigm shift in the principles and practices of many computer-based tools used in clinical practice [9].

## Level 2: Partial Automation

Partial automation in radiology refers to the use of computer-assisted diagnostic modalities to automate prioritization. However, the automation at level 2 requires radiologist supervision, and the diagnostic decision is not final without the radiologist’s approval. With the advancement of picture archiving and communication systems technology, radiology practices frequently consider upgrades and renovations to further improve efficiency. For example, radiomics is an emerging subfield of machine learning that converts radiographic images into mineable high-dimensional data by providing additional features to analyze and characterize the disease. Machine learning algorithms can be used to extract features from radiographic images that can help make prognostic decisions [10]. Feature extraction includes the texture, intensity, shape, and geometry of the region of interest [11]. Besides feature extraction from images, clinical and molecular profile data could sometimes be essential to comprehend complex diseases and ensure the right diagnosis to deliver the best possible treatment [12]. The amalgamation of machine learning, radiomics, and clinical information has the potential to improve its application in precision medicine and clinical practices. Since these technologies are still in their nascent stages of development, radiologists will most likely use them as ancillary tools in making final decisions.

The progress at this level of automation is slow and can be attributed to three major factors:

1. Lack of high-quality data: There is a limited amount of good quality medical data because the annotation and documented diagnosis by an expert are time-consuming and expensive processes [13]. This becomes a challenge when developing an AI system that can generalize well across unseen data, because the performance of machine learning models is significantly influenced by the size, characteristics, and quality of the data used to train them. The problem of insufficient training data, particularly in cases of rare diseases, can be addressed through data augmentation, in which synthetic data are generated to increase the prevalence of the target category, making the models more robust for analyzing independent information on the test sets [14]. Generative adversarial networks are the most commonly used neural network models for generating and augmenting synthetic images for rare diseases, such as rheumatoid arthritis and sickle cell diseases. Although these techniques allow models to be trained on sparse data sets and produce promising results, the adoption of generative adversarial networks in medical imaging is still in its early stages [15].
2. Stringent data laws: Medical data are often governed by several data security laws, regulations, and compliances, making it extremely difficult to share and use this data outside a clinical setting [6]. Collaborations between hospitals and tech companies are critical to bypass the barriers of data-sharing laws and make the best use of rich medical data to develop advanced solutions for automated and accurate diagnoses.
3. Cost of technology adoption: Current algorithms for analyzing radiological scans are computationally resource intensive, which significantly increases the cost of adopting these technologies in clinical practices. Therefore, it is important to develop low-power and cost-effective solutions that can be easily adopted by medical organizations. Edge devices can be used as low-cost prescreening tools as they can be deployed remotely and deliver instant results without consuming much bandwidth [16].

## Level 3: Conditional Automation

Unlike partial automation, where the final decision is entirely dependent on the radiologist, the systems at *Level 3: Conditional Automation* are robust enough to diagnose and make decisions under a predefined set of conditions (ie, those used to train the model) without radiologist supervision. If these conditions are not met, a radiologist must be available to override the AI analysis. The efficiency of human-AI collaboration in clinical radiology is dependent on clinicians properly comprehending and trusting the AI system [17]. One of the major requirements to enable such human-AI interfaces in radiological workflows is an effective and consistent mapping of explainability with causability [18]. Specialized explainer systems of explainable AI (widely acknowledged as an important feature of practical deployment of AI models) aim at explaining AI inferences to human users [19]. Explainability in radiology can be improved by using localization models, which can highlight the region of suspected abnormality (region of interest) in the scan, instead of using classification models, which only indicate the presence or absence of an abnormality [20]. Although an explainable system does not refer to an explicit human model and only indicates or highlights the decision-relevant parts of the AI model (ie, parts that contributed to a specific prediction), causability refers primarily to a human-understandable model. Causability is the degree to which an explanation of a statement to a human expert achieves a defined level of causal understanding while also being effective, efficient, and satisfactory within the context of use [18].

Radiology scans often suffer from high interreader variability that arises when 2 or more readers disagree on the results of a scan. This may lead to uncertainty in the ground truth labels. The problem of ambiguous ground truth can be mitigated by using expert adjudication [21] or multiphasic review [22] to create high-quality labels, which may help yield better models than other approaches in improving model performance on original labels [20]. Additionally, imaging protocols, manufacturers of imaging modalities, and the process of storing and processing medical data differ between organizations, which impedes the use of data from different sources for AI applications [23]. These factors result in the development of AI systems on a limited distribution of data, making them highly susceptible to failure if certain conditions, such as demographics, race, gender, time, etc, are not met. For example, Dou et al [24] developed a COVID-19 detection model using data sets from internal hospitals in Hong Kong. The model performed extremely well in identifying abnormalities in Chinese data sets but underperformed in German data sets with different population demographics [24]. Cross-center training of the AI model for different demographics and distinct cohort features would help the model learn from multiple sources and mitigate the problem of generalizability.

## Level 4: High Automation

Advancing from level 3, the AI systems at *Level 4: High Automation* would make decisions without the assistance of a radiologist. Human intervention would only be required in complex cases where the AI requests it. Such systems would require extensive clinical validations before they could be reliably used. As summarized by Kulkarni et al [20]*,* these systems would need to undergo internal as well as external validations to evaluate the system’s performance on unseen data. The Transparent Reporting of a Multivariable Prediction Model for Individual Prognosis or Diagnosis (TRIPOD) statement [25] specifies guidelines for reporting the development and validation of such diagnostic or prognostic models. Since these AI systems must work independently of conditions, they must generalize across a wide variety of data from different sources without inducing any bias from the training data. For example, Obermeyer et al [26] exposed a shortcoming in a widely used algorithm in the health care system that identified Black patients as being healthier than equally sick White patients. The racial bias exhibited by this system led to an unequal distribution of health care benefits to Black patients.

Elgendi et al [27] observed that adopting simple data augmentation and image processing techniques such as normalization, histogram matching, and image reorientation can aid in standardizing images from different sources. The standardization of annotation, data processing, and storage protocols are also vital for this data to be efficiently used for the development of AI systems. To learn and understand the differences and nuances of abnormalities in images from different regions, these AI systems would need to be developed on radiographic image data from various sources around the world.

The sharing of medical data has its own logistic and legal challenges as several government policies and compliances such as the Health Insurance Portability and Accountability Act [28] restrict the cross-border sharing of medical data. This is where privacy-preserving distributed learning techniques such as federated learning [29] and split learning [30] could play an important role in training the AI models at the source without moving the data to a centralized location. In the current state of development, the adoption of these distributed learning techniques is challenging because of the high costs involved in software development and infrastructure maintenance at multiple locations [31]. Despite these challenges, distributed learning appears to be a viable and promising approach to develop AI systems on multiple centralized data sets without the egress of sensitive medical data [32].

## Level 5: Full Automation

*Level 5,* referred to as *Full Automation*, is the ultimate stage of automation in radiology, where an AI application would be capable of end-to-end analysis of a case, from the initial diagnosis to automatic report generation. With the standardization of diagnostic reporting protocols and the recent advances in natural language generation models such as Generative Pre-trained Transformer 3 [33], results can be automatically reported in a structured format.

With such a high level of automation, it is crucial to maintain these AI systems at their optimal performance levels; however, their efficiency often deteriorates over time [34]. This phenomenon is referred to as model decay. One of the reasons for model decay is covariate shift [35], where the distribution of the input data is different from the training data. Another reason for such a decay could be prior probability shift [36], where the distribution of the target or the prevalence of an abnormality in a population changes. The change in the definition of the relation between the input and target data, referred to as concept drift [37], could also contribute to model decay. These changes may occur gradually over time or suddenly when the AI system is deployed in a different location with a different population. Therefore, it is crucial to continuously monitor these AI systems and fine-tune them as required to maintain optimal performance [20].

The complete automation of radiology in clinical practice will be challenged by medico-legal concerns about assigning liability in cases of AI misdiagnosis. A challenging legal question is whether doctors, radiologists, and health care providers would still be held accountable to bear ultimate legal responsibilities when they are no longer liable for the interpretation of radiological studies or would the data scientists and the manufacturers involved in the development and implementation of AI systems be held responsible [38]. It is important to focus on ethical questions concerning the implications of full automation for patient-centered medical care. In any event, responsibility must be assigned to humans for their involvement in this extremely complex field of AI in medicine [39]. Another challenge at this stage would be to address the fear among radiologists of AI systems taking over their jobs [40]. However, jobs will not be lost, but rather, roles will be redefined. With the influx of new data, radiologists would be the information specialists capable of piloting AI and guiding medical data to improve patient care [41]. AI will undoubtedly be an integral part of medicine, particularly radiology, but the final decision will be made by human radiologists because only a human expert’s knowledge and subject expertise can enable a reliable diagnosis [42]. We believe that AI systems will become smart assistants for radiologists, capable of automatically performing mundane tasks, such as preliminary diagnosis, annotations, report generation, etc, under radiologist supervision. This will not only reduce the workload of radiologists but also allow them to collaborate with clinicians and actively participate in other aspects of patient care.

## Conclusion

The advancement in AI is bringing the field of radiology to a higher level of automation. We propose a level-wise classification system for automation in radiology to elucidate the step-by-step adoption of AI in clinical practice. We also highlight the concerns and challenges that must be addressed as radiology advances toward complete automation. This includes the development of AI models that are transparent, interpretable, trustworthy, and resilient to adversarial attacks in medical settings. Developers of AI algorithms must be cautious of potential risks such as unintended discriminatory bias, inadvertent fitting of confounders, model decay, the constraints of generalization to unseen populations, and the imminent repercussions of new algorithms on clinical outcomes.

There are numerous ethical issues and unanticipated repercussions associated with the introduction of high-level automation in health care. To address these issues, regulatory standards for the development, management, and acquisition of technology and AI; public-private institutional collaborations; and ethical and responsible application of AI in the health care sector are required [43]. Most people envision AI fully replacing the driver or completely bypassing the doctor when they think about complete automation in the automobile or health care industry, respectively. Although there could be many good reasons to entirely replace drivers with autonomous vehicles, this approach could be detrimental in the health care sector. We must acknowledge the distinct advantages of augmentations over complete automation in health care practices [44]. In this regard, “expert-in-the-loop” ideology facilitates the collaboration between AI scientists, software developers, and expert radiologists. This substantially improves the quality and quantity of expert clinical feedback and guidance at every stage of development. As we move closer to the complete automation of radiological analysis, such collaborations are crucial for expediting the automation process.

## Abbreviations

AI: artificial intelligence
TRIPOD: Transparent Reporting of a Multivariable Prediction Model for Individual Prognosis or Diagnosis
